# Evidence of the Earliest Thyroid Cancer Operation Performed Successfully

**DOI:** 10.3390/cancers14143389

**Published:** 2022-07-12

**Authors:** Niki Papavramidou, Ourania Kalogeridou, Theodossis Papavramidis

**Affiliations:** 1 Museum for the History of Medicine, School of Medicine, Aristotle University of Thessaloniki, University Campus, GR-54124 Thessaloniki, Greece; npapavramidou@auth.gr (N.P.); rkalogeridou@auth.gr (O.K.); 2 1st Propedeutic Department of Surgery, AHEPA University Hospital, School of Medicine, Aristotle University of Thessaloniki, GR-54124 Thessaloniki, Greece

## 1. Introduction

Thyroid surgery appears early in history, even though the anatomic description and the function of the thyroid gland was not understood. Aulus Cornelius Celsus (1st c. AD) describes “bronchocele” as a growth existing in the neck, between the skin and the trachea, containing various kinds of material that is always enclosed in a coat, probably corresponding to a cyst. He proposes the surgical removal of the growth as the quickest method of treatment [1]. Aetius (5–6th c. AD) is another medical author who is often mentioned when tracing the history of thyroid surgery. Although he refers to five types of growths of the area (malleable, fatty, lumpy, scirrhous, and cancerous), he proposes the use of surgery only for the treatment of the first three, without describing the operation [2]. Paulus Aegineta (7th c. AD) also refers to growths in the neck, either steatomatous or aneurismatical, suggesting the use of surgery only for the former one, again without clear description of the operation [3].

Considering that thyroid cancer was differentiated from goiter in the 19th century, it is extremely difficult to trace back in early history to find the answer to whether surgical attempts were actually performed to cure thyroid cancer or goiter. Such a task is only made possible when a detailed description of the growth, clinical manifestations, and/or postoperative course are provided by the early authors. This paper seeks to trace back in history to find the first accurately described attempt of surgical removal of a thyroid cancerous growth, based on textual proof and solid data.

## 2. Materials and Methods

The quest for undoubtable data in the early history of medicine is always problematic. Primary textual sources dating before the 19th century are almost always vague, not very detailed and full of unexplained conclusions. The earlier the sources, the more unclear and ambiguous the descriptions of medical entities and methods are. While in the early texts we find references that probably relate to thyroid surgery such as in the cases of Celsus, Aetius, Paulus Aegineta, Albucasis, and Roger Frugard [1,2,3,4,5], it is impossible to investigate whether these accounts refer to cancer or simple goiter. Taking into consideration that Andreas Vesalius established the thyroid gland as an anatomical organ in 1543, while Thomas Wharton named and fully described the gland in 1656, this inability is justified. We thus focus on authors dating after the 16th century, accessing their primary texts and descriptions. In almost all cases, the data were clear enough to understand whether the authors were discussing the surgical treatment of a goiter or of a thyroid cancer.

## 3. Results

Doing research on the first attempts of thyroid cancer surgery has two great problems. First, there is no differentiation between goiter and cancer in the texts; this differentiation started to be more concrete during the 19th century, especially after Gaspard Laurent Bayle [6]. Second, the descriptions of the authors are not detailed enough to give sufficient information. An example of the latter is the case of Desault, who is widely recognized as the person who performed the first operation on thyroid cancer: in his text, we find indeed a first, this being the successful one-sided extirpation, but we do not read anything about the type of the growth that might give us a hint of a malignancy.

Instead, Mursinna’s postoperative description and especially the post-mortem examination provide us with all information required—for that time period—to safely presume that the patient operated suffered from a thyroid carcinoma. The clinical course of the patient (including rapid growth of the mass with pain and difficulty to breathe) in combination with the sudden death a few months after the operation make us think that this is probably an anaplastic thyroid cancer (poorly differentiated thyroid cancer).

## 4. Discussion

The 17th century discussion on surgical treatment of thyroid growths is dominated by Fabricius Hildanus, 1560–1634, famous anatomist and surgeon who authored, among other writings, 600 surgical observations and cures, similar to today’s case studies [7]. In this collection of cases, we find a description of surgical excision of a thyroid growth—probably goiter—performed by an empiric doctor, during which the patient died of excessive bleeding. What is interesting, though, is that he vividly describes how much surgeons feared this operation and attempted to avoid performing it due to the risk of bleeding and of the partial or total loss of the voice of the patient [8]. Johannes Dolaeus [9] and Peter Dionis [10], famous surgeons, royal physicians and professors of the same century, also describe thyroid surgery on goiters and not cancer.

The literature of the 18th century provides us with numerous descriptions of the surgical excision of goiter, such as in the cases of Gottfried Purmann [11], Heister [12] and Johann Astruc [13], while during this century we find Peter Joseph Desault’s description of the first successful typical one-sided extirpation [14]. What is of great interest, though, is the beginning of a “premature” discussion that differentiates simple thyroid growths from “malignant” ones, such as the analytical description of partial goiter extirpation provided by Adolf Friedrich Vogel, in his thesis entitled *Dissertatio inauguralis medica observationes quasdam chirurgicas complexa quam praeside viro illustro experientissimo ac celeberrimo*, published in 1771. We read:

“At times, however, it [the growth] displeases girls, especially in size and unconformity, to those who are more earnest in beauty, or, through their pain, threatens malignancy and even more serious dangers”.

Christian-Ludwig Mursinna, though, is probably the surgeon who first excised a malignancy of the thyroid. He was a German surgeon of the second half of the 18th and the first two decades of the 19th century. He was born in 1744 and, at the age of 13, he became an apprentice of a surgeon in Colberg. His apprenticeship coincided with the three Russian occupations of the area, and his whole academic and professional course was marked by the turbulent European military history of those times. As early as 1761, he worked in field hospitals as a surgeon, a post he served in under several circumstances during his long career. His clinical experience having preceded his academic training, he nevertheless sought to complete his education on various occasions and at several institutions, such as Potsdam, Berlin and Jena, where he received his PhD in 1798. Having even worked as a barber early in his life, he worked as a battalion surgeon several times, as a surgeon and member of the teaching staff in the Charité, he published several medical works, issued a journal on Surgery and Medicine and organized field hospitals. He died in 1823, having celebrated his 50-year anniversary in working as a surgeon.

He described a very interesting case that he came upon in 1787. A doctor noticed an unpainful growth on the front part of the neck of a patient that increased from month to month. Soon, it started producing pain and difficulty in breathing, and Mursinna was summoned to excise the growth. Upon examination, he found a growth of the size of a clenched fist and he performed the operation. Bleeding was profuse, but numerous doctors were present and “jumped in and squeezed the many wounds in the arteries with their fingers, and I calmly eliminated the whole tumor”. The wound healed well, and the patient had no pain or fever. However, 3 months after the operation the patient died suddenly, appearing to suffocate, and Mursinna asked for a post-mortem examination to learn why. The operated site was clean, but numerous growths were found between the pharynx and the trachea and in the esophagus, while the lungs were full of hard lumps firmly attached [15].

## 5. Conclusions

This article highlights the key role that Christian-Ludwig Mursinna played in the history of thyroid cancer surgery. We provide the description of a surgical operation of an anaplastic thyroid cancer, accompanied by a postmortem examination, clearly pointing at that direction. We should pinpoint the importance of primary sources when working on the history of medical achievements. It is very unfortunate that we very often take for granted information that spread from one author to another, ultimately reaching a bibliographical dead-end.
